# Access to and acceptability of sexual and reproductive health, harm reduction and other essential health services among people who inject drugs in Durban, South Africa

**DOI:** 10.1186/s12954-024-01042-6

**Published:** 2024-06-26

**Authors:** Cecilia Milford, Tammany Cavanagh, Shannon Bosman, Michael Wilson, Jennifer Smit, Brian Zanoni

**Affiliations:** 1 Department of Obstetrics and Gynaecology, Wits MRU (MatCH Research Unit), Faculty of Health Sciences, University of the Witwatersrand, Durban, South Africa; 2 Centre for the AIDS Programme of Research in South Africa (CAPRISA), Durban, South Africa; 3 Centre for Community Based Research, Human Sciences Research Council, Pietermaritzburg, South Africa; 4 Advance Access & Delivery South Africa, Durban, South Africa; 5 Department of Health Behaviour, University of North Carolina Gillings School of Global Public Health, Chapel Hill, NC USA; 6 Emory University, Atlanta, GA USA; 7 Children’s Healthcare of Atlanta, Atlanta, GA USA; 8 Rollins School of Public Health, Atlanta, GA USA

**Keywords:** Access to services, Harm reduction, HIV prevention and treatment services, People who inject drugs, Sexual and reproductive health, South Africa

## Abstract

**Background:**

People who inject drugs (PWID) are at risk of HIV acquisition. The number of PWID in South African cities is increasing, and in spite of an advanced HIV prevention and treatment programme, there are PWID who experience challenges accessing sexual and reproductive health (SRH) and HIV related services. Access to and acceptability of SRH and harm reduction services by PWID needs to be further understood and explored.

**Methods:**

In-depth interviews (IDIs) were conducted with 10 key stakeholders and 11 PWID, in Durban, South Africa. Interviews were transcribed and translated. Data were thematically analysed using Dedoose software.

**Results:**

Participants described stigma/discrimination from healthcare workers and other clients accessing services as barriers to accessing healthcare services. They were concerned about long waiting times at healthcare facilities because of possibilities of withdrawal, as well as lost opportunities to “hustle”. Targeted, non-discriminatory services, as well as mobile clinics existed in the city. Non-governmental organisations reportedly worked together with the public sector, facilitating access to HIV and TB prevention and treatment services. There were also needle exchange programmes and a harm reduction clinic in the city. However, there was limited access to contraceptive and STI services. Although there was reportedly good access to HIV and TB and harm reduction services in the city of Durban, uptake was low.

**Conclusions:**

The integration of services to enable PWID to access different services under one roof is critical. There is also a need to strengthen linkages between public and private healthcare, and ensure services are provided in a non-discriminatory environment. This will facilitate uptake and access to more comprehensive SRH and harm reduction services for PWID in Durban, South Africa.

## Introduction

Efforts to reduce the spread of HIV in South Africa have largely overlooked marginalised, high-risk populations, such as people who inject drugs (PWID) [1]. South Africa has the largest number of people living with HIV (PLHIV) globally, and extensive national programmes have seen some success in decreasing the overall number of new infections [2]. However, this downward trajectory applies to the wider, low- to moderate-risk population, which has historically been the focus of public health efforts to combat the disease [3]. High-risk, smaller, key population groups have recently been recognised as disproportionately contributing to the transmission of HIV and therefore requiring special attention [3]. These key populations, whose commonalities put them at higher risk of acquiring HIV than the general population, include various groups including sex workers and PWID.

There is limited to no reliable data on the size of the PWID population in South Africa [4, 5]. However, a recent (2020) estimation of approximately 82 500 [6], is higher than previous estimates: 75 700 [7] and 67 000 [8]. Although there are many contexts in South Africa where narcotic drugs are smoked or inhaled, intravenous injection (most commonly of opioids) appears to be increasing [1, 4, 9, 10]. Injecting drug use (IDU) has been shown to increase the risk of contracting blood-borne diseases like HIV and Hepatitis C [11], the likelihood of contracting HIV is 35 times higher for a PWID than for someone who does not inject drugs [2].

In South Africa, most people who inject drugs use a cheaper, low-grade heroin, which may be mixed with various substances [12], known locally as “whoonga” in KwaZulu-Natal (KZN) Province and “nyaope” in Gauteng Province [13]. PWID are at increased risk of acquiring HIV through unsafe drug use practises like sharing needles and “cooking” equipment (to mix and heat drugs) [14], as well as “bluetoothing” – the act of voluntarily injecting blood from someone who has just injected drugs into a person who has not used so that person may obtain a less-potent, secondary high [15–17]. PWID are also vulnerable to HIV infection through unsafe sexual practises [14, 18], such as unprotected transactional and non-transactional sex [19], as well as sexual violence and rape [13]. Amongst 450 PWID sampled in 2013, half had used a condom during last sex, and just under half had shared injecting equipment when last injecting [19]. Therefore, it is vital that PWID have adequate, acceptable and consistent access to HIV prevention and other healthcare services, including sterile injecting equipment.

A study in South Africa (in 2018), demonstrated low uptake of HIV testing services nationally, with only 24% of people being tested for HIV over that period, although results were slightly higher in Durban, where 31% had tested for HIV [20]. In that study, 33.3% of PWID nationally tested positive for HIV, but only 9% in Durban tested positive [20]. In South Africa, the proportion of PWID testing positive for HIV seems to be increasing, from an estimated 14% in 2013 [1] to a recent study reporting that 21% of PWID sampled had acquired HIV [5]. A global review of HIV intervention coverage among PWID estimated that in sub-Saharan Africa, only 1% of PWID had been tested for HIV [21], indicating a scarcity of service uptake and/or provision in the region – although data are lacking. In the South African study, only 14% of people with HIV nationally were on antiretroviral treatment (ART), and only 6% in Durban [20]. Indications suggest that HIV infection amongst PWID will continue to increase without intervention [3]. This hidden epidemic must be addressed to avoid substantial implications for the public health system. Understanding the health-seeking behaviours of PWID and investigating the resources available to them is therefore essential.

In order for the recent gains in tackling the HIV epidemic in South Africa not to be undone, equitable access to and engagement in HIV prevention and care must be realised across all sectors of society. Stigma and discrimination associated with being “*amaphara”* (derogatory term to refer to a criminal/drug user) [22], are cited as reasons for under-utilisation of public healthcare services by PWID, and they may rely on civil society organisations for healthcare needs over public sector services [13]. In South Africa, HIV prevention and treatment programmes which target PWID are limited in number, location, consistency and funding [17].

To investigate patterns in how PWID access and engage in healthcare behaviours in a localised South African context where there is high HIV prevalence, we explored access to and acceptability of sexual and reproductive health (SRH), HIV prevention and treatment, and harm reduction services among PWID living in the city of Durban, KZN Province.

## Methods

This qualitative research was part of a pilot study estimating the acceptability and feasibility of using respondent driven sampling (RDS) to access and enrol PWID in Durban, KZN, South Africa (perceptions of this methodology of recruitment will be published elsewhere). We piloted RDS with 45 PWID and conducted a survey with the identified individuals to learn more about their vulnerability and the influence this has on the HIV continuum of care in South Africa. Eleven of these PWID were purposively selected and invited to participate in in-depth interviews. In addition, key informants were invited to participate in an in-depth interview to explore these issues. COREQ (consolidated criteria for reporting qualitative research) guidelines were used to report the research process below [23].

### Study population and data collection processes

#### Key stakeholders

In-depth interviews (IDIs) were conducted with 10 key stakeholders working with PWID in various capacities, at national, regional and local levels in South Africa. Stakeholders were purposively selected and invited to participate in the research, and some stakeholders recommended interviewing additional key stakeholders with relevant expertise. There were no refusals to participate, only recommendations for more suitable participants. The stakeholders had various roles, including representatives from NGOs providing targeted healthcare and/or harm reduction services (*n* = 5), representatives from advocacy organisations representing PWID and/or sex workers (*n* = 4), and a relevant government department representative (*n* = 1). We stopped recruiting stakeholders once saturation was met and no new information was being gleaned through the interviews.

These semi-structured IDIs were conducted prior to contact with any PWID, during the period October 2020 to March 2021. Key thematic areas explored strategies to recruit PWID in research, as well as information on access to SRH and HIV related services for PWID, specifically in Durban and surrounds.

All stakeholder participants provided written informed consent. Interviews were conducted remotely (via telephone/Zoom) or in-person, depending on participant preference and COVID-19 protocols at the time. IDIs were conducted in English or *isiZulu*, depending on participant preference, and lasted between 1 and 1.5 h. Interviews were conducted by trained female researchers with degrees/advanced social science degrees (CM, TC and one other trained researcher, fluent in *isiZulu*). In most cases, there was no prior relationship with key stakeholders, although one participant (an advocate for sex workers) was known to the researchers.

### People who inject drugs

Following the stakeholder IDIs, contact was initiated with the PWID community through a harm reduction centre operating in Durban. Using RDS, 45 PWIDs were recruited to participate in a survey, from November 2021 to February 2022. All PWID were over 18 years of age, and self-reported injecting drugs in the last 6 months. The survey included questions on demographics, health service access, drug/alcohol use, HIV prevention, HIV and Hepatitis testing, HIV positivity, etc. Surveys were conducted in *isiZulu*, in a private interview room at a research centre, by female interviewers trained in quantitative and qualitative data collection, with training in collecting sensitive data from vulnerable participants. The interviewers were not known by the participants. Data were directly entered into REDCap [24] by the interviewers during the interviews, and the process took about an hour.

Eleven PWID were purposively sampled from the RDS survey participants and invited to participate in an IDI during the period December 2021 to February 2022. IDIs were conducted on a different day from the survey, at a time convenient to the participants. The purposive selection was to ensure representation of both male and female participants, in various stages of the RDS survey network. There were no refusals to participate in an IDI. Similar to the key stakeholder IDIs, IDIs with PWID were stopped once saturation was met. All IDIs were once off and were conducted one-on-one, in-person, in a confidential setting at the research centre. They were all conducted in *isiZulu*, the preferred local language, and lasted between 1 and 2 h. The IDIs were conducted by the same female interviewers who conducted the survey research. These IDIs explored factors including PWID’s access to and use of SRH and HIV prevention and treatment services, and the impact of COVID-19 on access to services. The IDI guides for PWID were not the same as those used for key stakeholders, although similar thematic areas were explored.

### Data analysis

The survey data describing drug use behaviour and access to healthcare services were quantitatively analysed, and have been reported elsewhere [17].

All IDIs were audio recorded, with participant consent, transcribed and translated (where necessary) into English. Transcribers were fluent in both English and *isiZulu*, and interviewers also assisted, either in review or transcription of the interviews, to ensure accurate transcription of the IDIs. Following transcription, a single code list was developed using both inductive and deductive strategies, based on a review of transcripts from IDIs conducted with both key stakeholders and PWID. The codelist was informed by thematic areas arising from the data and influenced by the structure of the questions. Two researchers (CM and TC) read the transcripts and through repetition, identification of similarities and differences, and interpretation of data, thematic areas were identified and a codelist was developed [25]. This was reviewed and discussed by CM, TC and BZ. Once agreement was reached, CM and TC each coded a portion of the data until all transcripts were coded. Dedoose software (version 9.0.62, LA, California), was used to organise, code and facilitate analysis of data. Thematic areas are described in the results section below, and illustrative quotes have been identified to highlight key thematic areas.

### Ethical considerations

This study was approved by the Human Research Ethics Committee (HREC) of the University of the Witwatersrand (Reference: 200,202) and the Emory University Institutional Review Board (IRB) (ID: STUDY00000134). All participants (key stakeholders and PWID) were 18 years or older, and provided written informed consent to participate in the survey and/or IDI, with separate consent for audio recording of their IDIs. Consenting was conducted in *IsiZulu* or English, depending on participant preference.

## Results

### Socio-demographics

There were 10 key stakeholders and 11 PWIDs who participated in IDIs. Key stakeholders included representatives of non-governmental organisations (NGOs) providing healthcare services to PWID, advocates for sex workers and/or PWID, and national government representatives with a mandate to work with substance abuse. The key stakeholders ranged in age from 29 to 65 years. There were 7 female, 2 male and 1 gender non-conforming key stakeholder participants.

There were 6 female and 5 male PWID participants, who ranged in age from 21 to 36 years at time of IDI, with a median age of 28 years. The median highest level of education of the PWID participants was grade 10, with a range from grade 7 to tertiary level education. Although all PWID participants (*n* = 11) reported that they had been homeless in the past year, seven reported that they were currently homeless. Only 3 PWID participants reported that they were currently unemployed, and the majority reported part-time employment (*n* = 5) – including examples such as sex work, washing cars, and “hustling”. Two reported that they were self-employed (one street vendor, and one did not specify type of employment). Ten of the PWID participants had previously tested for HIV, and only one reported that they were living with HIV.

### Thematic findings

Both key stakeholder and PWID responses are reported below – any differences in opinions are highlighted, if there were no differences, results are summarised. Information is presented according to the following thematic headings: Barriers and enablers to accessing healthcare, existing healthcare services, harm reduction and needle exchange services, HIV testing and treatment attitudes and practices, PrEP attitudes and practices, and suggestions to meet the SRH needs of PWID.

### Barriers and enablers to accessing healthcare services

Various barriers and enablers were reportedly experienced by PWID when accessing general healthcare services.

#### Barriers

The *discriminatory behaviour* of healthcare workers towards PWID was highlighted as a major barrier to accessing healthcare services. Discrimination from others, including security personnel and other clients accessing services, was also described. It was felt that PWID were particularly stigmatised for a number of reasons over and above their use of drugs, including homelessness, possible sex work and criminal behaviour/s.

*[A]lso I’m complaining [about] security guards sister, they are the ones that send people away. I won’t complain about the people inside [referring to healthcare providers]. […] They don’t know what happens at the gate, it’s the securities. […] Before you enter […], the securities they say, “go away, get away from here”.*** PWID, Female, 21 years**.

*They discriminate us in those clinics if you are homeless, you do not get help.*** PWID, Male, 36 years**.

*[B]ecause of the stigma attached to it, so if someone knows that the doctor’s going to try and take their blood pressure and looks at their arms, you know, that fear is there, they’re going, the likelihood of them being told, “why are you injecting” or, “no you did this to yourself” or all those lovely discriminatory statements.*** Key stakeholder, Female, 29 years**.

*Long waiting times* at public healthcare facilities were also a barrier to accessing healthcare services, largely because of fears of going into withdrawal whilst waiting, as well as the missed opportunity to make money or “hustle”.

*The challenge for clients going to clinics and things like that is the long waiting periods and them going into withdrawal ‘cause they fear the withdrawal they would maybe go to the clinic but then leave to go and earn money, or skurrel [meaning hustle for money], or use, and then not return. And that, that is a big challenge.*** Key stakeholder, Female, 44 years**.

*[A] phara [homeless person] won’t stand on the queues and waste their time. Because at the clinic you stay whole day there. […] Oh my God, when would you hustle?*** PWID, Female, 27 years**.

The discrimination experienced and fear of waiting in queues, as well as lack of motivation experienced by PWID, reportedly led to *delayed access to healthcare services*, and consequently *poor health outcomes*.

*You cannot go to the clinic in the morning for [health care services] and wait until the afternoon. So, they get very lazy to go there because at times you find that one ends up dying, something that could have been avoided if they had gone to the clinic for help. So, they end up not going to the clinic to deal with their health.*** Key stakeholder, Female, 53 years**.

*At the clinics it just that most of the time we don’t like going there because you feel isolated you see, it is the way they are treating us, they don’t treat us well as normal people you see, that is why some of us prefer dying not taking their pills because we don’t feel comfortable going to the clinics.*** PWID, Female, 21 years**.

#### Enablers

Various factors were identified as enablers to accessing healthcare services in the Durban area. Targeted, non-discriminatory services, closely located, with limited waiting times and extended operating hours were valued.

*I like a place that is operational for 24-hours the whole night, staff has to take turns because if I have a problem at 1 in the morning when they have closed, […] if it is a clinic then I understand it must close, but the hospital must not close, until then when I need help I must be able to get, I like such places.*** PWID, Male, 26 years**.

All PWID had accessed healthcare services from providers who were supportive and non-discriminatory, the majority of whom worked in private/NGO targeted healthcare facilities, although a few PWID described positive experiences from healthcare providers at government/public healthcare facilities.

*What I can say is that when people are at work they are not the same. There are some that treat people well and some treat them otherwise and when you say something they will not even have time to listen to you. Personally I rate highly [name of public hospital], it is the best […]. They treated me well, I am now referring to hospitals now not clinics. Whether you are homeless or what they don’t care they treat you the same as equals.*** PWID, Male, 26 years**.

Mobile clinics/outreach services were viewed as enablers to accessing healthcare services, for example HIV counselling and testing (HCT) services.

*Where you find I’m just walking in the street, out of blue, here’s a tent, I then test for HIV.*** PWID, Male, 30 years**.

Providing integrated services within a caring environment was seen as an enabler to accessing treatment and facilitating adherence.

*[A]t [name of area] it is better because there are sisters [nurses] who do give them medication, they give them food first then give them medication.*** PWID, Female, 28 years**.

### Existing healthcare services

Key stakeholders and PWID described various targeted/dedicated healthcare services operating in the Durban area, targeting homeless people, sex workers and/or people who use drugs (PWUD) -providing services more broadly than only for PWID. Some of these services had drop-in centres, others had mobile clinics or outreach teams, or both. The targeted services were largely operated by NGO/private funding, but were reportedly working closely together with public healthcare services/Department of Health (DoH).

A variety of healthcare services were provided as key/core services by the different targeted service providers. For example, core services included primary healthcare for homeless people, harm reduction services (needle exchange or methadone programmes), and/or HIV and TB testing and screening services. PrEP, ART initiation and TB treatment services were also available. However, not all targeted service centres provided all these services, instead some facilitated referral/linkage services to access any additional services. Partnerships and referral relationships between different NGOs, private sector and DoH services – where private sector and DoH reportedly provided medicines (e.g. for TB and HIV), and NGOs had set up systems of delivery of these medicines to minimise loss to care - were described. As a result, almost all PWID described that they were able to access some healthcare via these targeted services.

*Because [name of targeted healthcare centre] is a place […] for people like us. They have a way to take people in, […] So they also have a way to treat people. Even if you maybe came in because of being sick, they treat you nicely, ask you, “have you eaten?”, they tell you, “do not take pills before you have eaten”, then you can see that this is a free person.****PWID, Female, 21 years***.

There were also reportedly some psychosocial services available to PWUD, whereby some NGOs provided group/individual counselling sessions on select days of the week. However, a key stakeholder described that they were “*advocating for more psychosocial services to be made available and easily accessible*” (Key stakeholder, Female, 29 years) to PWUD.

However, there was reportedly limited access to contraceptive services, pap smears, STI testing and screening and termination of pregnancy in the targeted services.

*[W]e don’t offer contraceptives, [and] they are not willing to go to the facility to get contraceptives because the nurses discriminate against them […], “oh you a drug user why you even coming?”*** Key stakeholder, Female, 31 years**.

A stakeholder working in a facility offering targeted services described how this was overcome.

*[I]f a client needs immediate medical attention or needs to be referred for ART treatment or TB treatment through our linkage officers we escort the client to our engaged or referral health facilities. We take the client to be initiated onto treatment, we do a second follow up, third follow up, until the client is comfortable to wanting to go to the facility on their own.***Key stakeholder, Female, 31 years**.

### Harm reduction and needle exchange services

Although a few drug rehabilitation/detox centres were listed as available in the area, key stakeholders described how there had been a recent move from the abstinence model of treatment to harm reduction services. In spite of some challenges accepting harm reduction as an approach in the general population, key stakeholders and some PWID viewed harm reduction services as beneficial.

*[W]hat research shows is that those on maintenance therapy for 6 months or more, are more likely to stop their substance use, and return to kind of living normal life etcetera, than those that are not on maintenance. So you would find that a lot of heroin users go on detox and relapse.*** Key stakeholder, Female, 44 years**.

*[I]f you inject perhaps let us say 7 times a day, they will tell you to reduce down at least to inject 3 times so that because they are trying to drive you to want to think of washing and bathing, and now the way things happen is that the whoonga… there is this thing called methadone, methadone doesn’t prevent cravings for whoonga but it is there to help you think of other things around such as washing, bathing and be clean, they are actually implying that you can do drugs but you mustn’t look like you are no longer people and you are dirty.*** PWID, Male, 26 years**.

PWID and key stakeholders described a harm reduction centre, providing methadone, operating in the city centre. In addition, methadone was reportedly accessible through a separate NGO – although the linkages between the two facilities were not well described. The harm reduction centre had been recently established, facilitated as a result of drug withdrawal that had been experienced by people residing in homeless shelters set up during COVID-19 lockdown period.

*[S]o for them, it’s like they say “Thank God to lockdown”, you know, because now we have a safe-, a safer place, we have a methadone treatment, and we were taken to a place of safety, but before, we were seen as street kids, you know, as “paras,” or people, didn’t really care about us, unless they were involved in anything that you do like, you know organisation that works with key populations, but everybody, the City, the municipality of eThekwini [Durban] got, got involved, and the DSD [Department of Social Development] got involved, so they say they see a lot of improvement, and they say “thanks to lockdown that happened”.*** Key stakeholder, Female, 44 years**.

The harm reduction centre was described favourably - in addition to providing methadone, it provided access to sterile needles, as well as to chronic medication such as HIV and TB treatment services.

There were reportedly a few needle and syringe exchange programmes operating in the city at the time of the study – largely provided through a Global Fund funded NGO. Most PWIDs described accessing free needle and syringe packs (and disposal containers) through this NGO, often through mobile/outreach support services. New packs were supplied to PWID when the used ones were returned.

*They give us…actually this company is saying the one that gives us needles, they saying if you finished with these needles you should put them in these containers, they gave us these safe containers you put it inside, if you have putted 25 when you bring it back to the scale they have and put this, the scale we tell how many needles and they will give you what equal to what you bringing.*** PWID, Male, 36 years**.

In spite of access to these needle exchange programmes, there was concern that sterile needles were not accessible daily/consistently to PWID, which could result in the reuse of/or use of unsterile needles.

*So my big challenge [is] they have two days that they are in Durban and it’s between certain times, […] you are not going to reach everyone during 2 h on 2 days. […] Ideally you need somewhere that they can go to daily.*** Key Stakeholder, Female, 30 years**.

*[Y]ou’d find that they would come and give us enough needles, and you will find that for three weeks they wouldn’t come, obviously that time we will be using the same needles and some people will be using them also, someone will come and borrow a needle.*** PWID, Female, 28 years**.

Furthermore, law enforcement was described as a challenge with needle exchange programmes – whereby any needles were confiscated, resulting in some PWID not wanting to keep their used needles for exchange.

*[W]e distribute a whole lot of needles […] the biggest problem is law enforcement, so what happens is that they confiscate those needles, or they stand on them, or they arrest them because they have them on them […] and that goes for having sterile needles on you, or used needles, so it’s not just used needles. […] And it also makes the return of those needles very tricky, because people don’t want to keep used needles on them and in order to have a sort of adequate robust needle and syringe programme, you have to get your needles back as well so that they are not going to reuse them.*** Key stakeholder, Female, 40 years**.

A key stakeholder described that the colour of the syringe caps on needles provided through the NGO was different from those available in the public sector, so that safe disposal could be monitored.

*[W]e changed the colour of our needles so that we account, our needles are now pink, so they not the standard orange ones, you know those diabetic needles you get? They’re not the standard, and that helps us to track, you know, what we’re responsible for and what we’re not.*** Key stakeholder, Female, 44 years**.

A few PWID described that they purchased sterile needles from people who had accessed them from needle exchange programmes.

*Interviewer: [W]here do you usually get your needles from, you said you get them from the bin?*

*Participant: Ja. And from my other brothers those who sells them, they get them from [NGO/private organisation], and I am not sure where they get them.*** PWID, Male, 32 years**.

### HIV testing and treatment – attitudes and practices

#### HIV testing behaviour

Ten of the PWID interviewed had tested for HIV in the past – many describing regular testing at mobile clinics or targeted services. Key stakeholders also described the benefits of providing HIV testing services in the community.

*I know it sounds crazy but, sit down under a tree, one-on-one with someone and do an HIV test. Sit down under a bridge and do an HIV test, and if someone is positive, have a nurse with me who can draw the bloods and put it in a cooler box and take it back to the office where it can then go for like CD4 counts and viral load and so forth. And, yes, I say again, it’s more work for the team but it makes… the testing, the counselling, the next steps easier because then also you’ve identified that person now.*** Key stakeholder, Female, 29 years**.

Although many PWID felt that it was important to know their HIV status, some were still scared of finding out their test results. One participant said that in the past she had not cared about her HIV status, but that through targeted services, social workers were encouraging PWID to get tested for HIV.

*[I]t was me who was hesitating [to be tested for HIV] because I know my ways. I was a little bit scared that perhaps there will be a problem [meaning be HIV infected], but when I start testing and find out that I was negative, that gave me strength and courage to come back when I have slept with someone I do not trust I have to go back for HIV testing because things they have explained to me, encourage me that if you go for testing it is nothing big because even if you positive it’s nothing big.*** PWID, Male, 36 years**.

### HIV treatment services

Only one PWID said that she was accessing HIV treatment services. She was also on methadone, and described how she was able to collect her methadone and ART from the harm reduction centre daily. Other PWIDs also described this integrated service delivery method as an enabler for people with HIV to access their treatment.

*Even those ones that have TB they take their pills every day, also the ones that are infected with HIV they take their pills every day at [harm reduction centre] so, something that social workers did there is that everyday they made us to go there daily. We go there everyday to take the treatment because it is well known that some of us will not just take their pills under the cravings and having [withdrawals], never.*** PWID, Female, 27 years**.

In addition to the harm reduction centre, other targeted service NGOs also reportedly provided support to PWID to access HIV treatment, either through linkage/referrals, or through taking treatment to community members.

*[I]n terms of ART we accompany, our linkage officers accompany clients to the facility […] in order to ensure that they are seen.*** Key stakeholder, Female, 44 years**.

*[T]he best way to address that is to meet them where they’re at. People have a routine and once we build up a routine of “cool, every Monday we go here, we provide ARVs here. Every Tuesday we go here, we do HIV testing and ARVs here”. And literally Monday through to Friday or whenever and that stays, come hell or high weather, that stays. Um, because again, going back to what I spoke about earlier is some of the community members don’t have access to a phone or to even a calendar and stuff but they do know “cool, today’s Monday, um, er, the group comes on Monday, Wednesday, Friday, great.*** Key stakeholder, Female, 29 years**.

Challenges to HIV treatment adherence were reportedly related to various factors, including personal challenges remembering to take antiretrovirals (ARVs), not wanting others to see them taking ARV tablets, not having access to food to take with treatment, or challenges with storing medication/getting it confiscated by the police.

*I think most of my friends who are positive do know, but they, I don’t think they adhere to treatment, because most of the time they will be, maybe, high to remember that they should take treatment, and then they end up defaulting.*** Key stakeholder, Gender non-conforming, 35 years**.

*Because of living on the street, or it being confiscated by the police, or not wanting your, […] the person next to you to see you take it, or see it in your bag, or your bag getting stolen by somebody else. It’s very hard to adhere to medication.*** Key stakeholder, Female, 40 years**.

### Oral PrEP – attitudes and practices

Most PWID had either heard about PrEP or been given oral PrEP for HIV prevention, and only one male PWID had never heard about PrEP. Injectable PrEP options were not discussed as, at the time, these were not available to the public. In spite of knowledge about oral PrEP, the majority did not fully understand/know how to take oral PrEP correctly. Some had only taken oral PrEP once because they did not realise it had to be used daily for HIV prevention, and one had discarded it because he did not actually want to use it. Only one female PWID was currently using oral PrEP.

*I was given a bottle so, for the lack of complete information or maybe like I did not hear well, then I took one pill thinking you only take one pill, you see.*** PWID, Female, 28 years**.

The majority of PWID thought that PrEP would be of benefit to others and that a daily pill would be acceptable to other PWID because of their HIV risk.

*I do not think there would be a problem to take pills every day. I do not think so.*** PWID, Female, 28 years**.

*They will be very happy [to start PrEP] because it easy to get infected with HIV when injecting because others you find that others […] do not have injection they will ended up using someone’s injection so that how they get infected with HIV.*** PWID, Female, 27 years**.

A couple of key stakeholders felt that PrEP should be provided as part of a “bouquet of services”.

*[M]y personal view is that PrEP is important, but it’s not the priority. The priority, HIV prevention intervention for people who inject drugs is access to sterile injecting equipment whenever they need it. And then PrEP should be offered as part of another bouquet of services if that’s part of what they require.*** Key stakeholder, Male, 40 years**.

Key stakeholders and PWID stressed the importance of education about PrEP to facilitate uptake and adherence, especially since they felt that many people had not heard about PrEP.

*A lot of education again is required […] because people don’t just agree to take something they don’t know. They want to know how it will benefit them.*** Key stakeholder, Female, 65 years**.

In spite of the perceived advantages of PrEP, there were concerns about challenges PWIDs might experience adhering to oral PrEP.

*[I]t’s a lot of these, the practicalities of taking medication. So if you live on the streets, your stuff gets stolen, you get harassed by the police, you have to move, the police steal your stuff, you know, people can have problems, or challenges, finding a routine of how to take medication if they’re not having regular times of ingesting opioids, but if people are guided to find ways to integrate their drug use with their ART use, they can be adherent and do very well. […] There’s no contraindication to heroin use and ART. There’s no contraindication to heroin use or methamphetamine use and PrEP.*** Key stakeholder, Male, 40 years**.

One PWID described how he would not take oral PrEP as he felt that it would be no different from taking ARV tablets.

*So basically I am not different from the person who has qhoks [HIV], the fact that I have to take pills even if I am not sick, you know that was what I did not like because I do not what to lie, immediately when I got them [referring to PrEP], I just threw them in the bin. I don’t think that thing worked for me.*** PWID, Male, 26 years**.

### Suggestions to meet SRH and HIV needs of PWID

Key stakeholders provided a few suggestions as to how to better meet the SRH and HIV needs of PWID. There were some suggestions for providing integrated services, and for creating appropriate linkage and referral between public and private sector services or agencies.

*[W]hat I’ve done in this current programme is that I’ve approached the facility through our professional nurse and I’ve asked the [Department of Health] facility if they can give us, instead of referring our clients to the facility, they will give us the ART medication and our professional nurse will initiate onsite. [….] And it’s working brilliantly.*** Key stakeholder, Female, 31 years**.

*I do think that integration is important within the mainstream services so that they are identifying and detecting, […], because people that come for sexual reproductive health services, for an HTS service don’t always come through the usual thinking of sexual reproductive health service, they come for an HIV test for some other reason.*** Key stakeholder, Male, 40 years**.

It was also described that targeted mobile or outreach services and peer supported services, provided sensitively and understanding the habits of PWID, would better meet their needs.

*But using, you know, secondary distribution of equipment and condoms and basic things should I think happen by peers that are capacitated and supported, and then, you know, mobile outreach services.*** Key stakeholder, Male, 40 years**.

*[T]here are probably are these windows of opportunity to deliver care to people, one where you know they are going to be more likely to receive, […] like physically actually receive the care, but then also be kind of motivated to stay on treatment etc., and it probably has to deal with when they last had their drug. […] and that seems to be a time when people would be more likely and more receptive to receive medical care.*** Key stakeholder, Male, 34 years**.

Finally, it was stressed that PWID need more support for ART treatment adherence than the general population, and that this could be done via sensitised counsellors and assisted medication storage.

*Their needs are different, absolutely, compared to the general population. They need more of a support in terms of adherence support, […] a lot of the times they’re discriminated against at the healthcare facilities and they refuse to go back. Unless of course they’re taken by (name of NGO participant works at) staff, or they go to a facility that’s already sensitised but even at the sensitised facility you do get some of the counsellor nurses that discriminate against them and so clients don’t wanna go back.*** Key stakeholder, Female, 31 years**.

*I think not having proper storage facilities, living on the street, medication getting stolen, I think what might make it easier is to work through NGOs like our drop-in centre for example where we could store the medication for them and they could get that daily. Ah, that’s one alternative. […], their meds are always going missing.*** Key stakeholder, Female, 44 years**.

One key stakeholder did however, express concern that focussing on the SRH needs of PWID would result in missing the SRH needs of other PWUD, and missed opportunities to prevent PWUD from progressing to injecting.

*So, by only focusing services on people who inject drugs you’re missing the people where you could actually intervene before they start injecting. […] so that’s like an important policy element and it’s got big implications around opioid treatment and, um, also sexual reproductive health services, it should not focus only on people who inject drugs.*** Key stakeholder, Male, 40 years**.

## Discussion

Participants in this study had high risk injection use practices, highlighting a need for access to SRH and HIV related services [17]. However, they reported various barriers to accessing healthcare services, especially from government supported public healthcare facilities. Barriers included discrimination experienced when accessing healthcare from public health facilities, multiple stigmas (because of homelessness, drug use and criminal behaviours), and long waiting times at facilities. Long waiting times are problematic when experiencing withdrawal and cravings, and further reduce time available for hustling to make money. This has been reported previously in South Africa [4], with PWID disclosing that they avoid hospitals, even when seriously ill, out of fear of experiencing withdrawal symptoms while there. The stigma and discrimination experienced results in inequalities accessing healthcare services [26] and has been reported previously [22, 27, 28]. Stigma and discrimination may affect surveillance data, and it has been speculated that HIV prevention and treatment programmes – as well as PWID themselves – may not report IDU in settings where it is illegal, for fear of social and legal repercussions [21]. Furthermore, poor access to healthcare services results in poor health outcomes and in extreme cases can lead to death, which has the potential to create additional burdens on the health care system.

In spite of the barriers, participants described some enablers to accessing healthcare services in the Durban area. They described existing targeted and/or dedicated healthcare services, including drop-in centres, mobile and/or outreach services. These services provided easy access to various non-discriminatory healthcare facilities, some with extended operating hours. Most of these targeted services were private or NGO supported services, and they reportedly worked closely together with the public health system, providing an array of services to PWID, many of which provided integrated services. Previous research has shown that PWID tend to rely primarily on “civil society organisations” over public health sector services for accessing healthcare [13], which was also found in this study.

Although these targeted services provided PWIDs with access to general healthcare services (including primary healthcare and HIV and TB testing and treatment), key stakeholder and PWID participants reported limited access to contraceptive services, pap smears, STI testing and screening and termination of pregnancy in targeted services in Durban. However, as has been reported elsewhere, in parts of South Africa, and in Durban more specifically, COVID-19 resulted in improved provision of care to PWID, especially through targeted and harm reduction services [17]. Participants described that harm reduction services were available in the district – in the form of methadone and needle exchange programmes [17, 20]. However, they also had concerns about limited daily access to sterile needles and potential confiscation of needles by law enforcement agents, resulting in some re-use and individual selling of needles. Furthermore, the needle and exchange distribution programme operating in the Durban area has been closed [29] and reopened in the recent past, causing concerns around reliability of access to sterile needles. Similarly, recent research conducted with PWID in South Africa reported that, in areas where no needle exchanges existed, PWID were forced to re-use and/or share needles [4]. The challenges accessing sterile needles need to be addressed at a programmatic level.

Although other research shows low uptake of HIV testing services by PWID [20], 10 of 11 PWID in this qualitative sample had previously tested for HIV, largely because of the targeted services and readily available testing sites in the city of Durban. One PWID participant self-reported as infected with HIV and reported she was accessing HIV treatment. This treatment was provided as part of an integrated service offered in the district (provided together with methadone), and such integrated treatment provision was viewed as beneficial by both key stakeholders and PWID. It was, however, felt that numerous challenges existed which would impact on ART treatment adherence for PWID, including remembering to take their treatment, not wanting others to see them taking treatment for fear of judgement, not being hungry or having access to food to take with their medication, and challenges storing and/or confiscation of medication due to homelessness [27, 30].

There is little data on PrEP and PWIDs in South Africa. In this study there was a focus on oral PrEP, since injectable PrEP was not yet available. Participants felt that PrEP would be acceptable and of benefit to PWID. However, there was little understanding about how oral PrEP worked or how it should be taken, highlighting a need for further education about PrEP in this population. In addition, both PWID and key stakeholders had concerns about daily oral PrEP adherence – describing similar reasons associated with ART adherence. Recent advances in the development of long-acting injectable PrEP mean that alternative PrEP products may be available to this population in future [31]. PrEP products which do not require daily dosing or adequate storage to maintain adherence, may be appealing to persons who are hindered by circumstances, such as PWID [27, 28, 30, 32]. Preventative treatments such as these may provide a more accessible and sustainable model of care for this key population.

Participants in this study provided numerous suggestions for improving access to healthcare, HIV and SRH services for PWID, and additional recommendations can be made making use of study findings. Many of these require radical programmatic solutions and support. Firstly, there is a need to improve linkage of PWID to appropriate facilities and provide them with non-discriminatory care and support. This can be achieved through peer supported and/or targeted services, which are provided by individuals or organisations who understand the specific healthcare needs of PWID. This care can be further facilitated through education of PWID about the availability of various treatment, care and support options, as well as through the appropriate sensitisation of healthcare providers and other staff working at healthcare facilities. The value of providing integrated services is also critical in addressing healthcare needs of PWID. For example, providing ART and methadone (or other opioid substitution therapy) together at a harm reduction centre, or food together with needle exchange programmes at an HIV treatment site, together with options for mobile healthcare services is recommended. There is a need to consider mechanisms to provide support for treatment adherence, for ARVs, PrEP, TB etc. For example, assisted medication storage could be considered. Finally, programmatic responses need to take into consideration regular and frequent access to sterile needles, possibly through needle exchange programmes that are supported by government and law enforcement agencies.

As with any research, limitations can be reported. This was a small qualitative study, so although findings describe access to healthcare and SRH services in the city of Durban, findings are not generalisable. Many of the described targeted services were/are provided via private and NGO funding, which is based on external funding support which can vary over time, and therefore may impact on the quantity and quality of services at any one time. Finally, the seed participants in the main study and some of their contacts were accessed via a harm reduction programme, and these participants may potentially be more likely to take care of themselves and follow up on health seeking behaviour than other PWID.

## Conclusion

In the city of Durban in South Africa, PWID in this study reported that they had good access to general healthcare through targeted and/or dedicated services. However, there was limited access to some SRH services, including contraception, STI screening and treatment and termination of pregnancy services. In this district there are high rates of unsafe injection drug practices, coupled with low uptake of HIV treatment and prevention services, and poor adherence to medication where treatment is accessed, putting this population at increased risk of HIV acquisition. There is therefore a need to strengthen linkages and referrals between PWID and appropriate healthcare services in order to facilitate access to these services. Furthermore, integrated service delivery could improve uptake and access to some SRH services. The success of the targeted services that are offered is linked to relationship building and trust between the PWID and the service providers, and also ensuring that the services provided are accessible in a non-discriminatory environment. In order to engage PWID in care, it is critical that the services provided are increasingly accessible and sustainable over time – something which integration of essential services and care may facilitate. Access to long-acting products that do not require storage, such as injectable PrEP, could add to sustainability. These research findings could influence future research directions and programmatic solutions, not only in South Africa, but also globally.

## Data Availability

Access to the data from this study may be requested through submission of a research concept to: cecilia.milford@gmail.com. The concept must include the research question, data requested, analytic methods, and steps taken to ensure ethical use of the data. Access will be granted if the concept is evaluated to have scientific merit and if sufficient data protections are in place.
